# First‐in‐human intracochlear application of human stromal cell‐derived extracellular vesicles

**DOI:** 10.1002/jev2.12094

**Published:** 2021-06-04

**Authors:** Athanasia Warnecke, Nils Prenzler, Jennifer Harre, Ulrike Köhl, Lutz Gärtner, Thomas Lenarz, Sandra Laner‐Plamberger, Georg Wietzorrek, Hinrich Staecker, Teresa Lassacher, Julia Hollerweger, Mario Gimona, Eva Rohde

**Affiliations:** ^1^ Department of Otorhinolaryngology, Head and Neck Surgery Hannover Medical School Hannover Germany; ^2^ Institute for Cellular Therapeutics Hannover and Institute of Clinical Immunology Hannover Medical School University of Leipzig as well as Fraunhofer Institute for Cell Therapy and Immunology (IZI) Leipzig Germany; ^3^ Department of Transfusion Medicine University Hospital Salzburger Landeskliniken GesmbH (SALK) and Paracelsus Medical University (PMU) Salzburg Austria; ^4^ Institute of Molecular and Cellular Pharmacology Medical University of Innsbruck Innsbruck Austria; ^5^ Department of Otolaryngology Head and Neck Surgery University of Kansas School of Medicine Kansas City Kansas USA; ^6^ GMP Unit Spinal Cord Injury & Tissue Regeneration Centre Salzburg (SCI‐TReCS) Paracelsus Medical University (PMU) Salzburg Austria; ^7^ Research Program Nanovesicular Therapeutics Paracelsus Medical University (PMU) Salzburg Austria; ^8^ Research and Transfer Centre for Extracellular Vesicle Theralytic Technologies Salzburg Austria

**Keywords:** cochlear implantation, EVs from umbilical cord‐derived mesenchymal stromal cells (UC‐MSC‐EV), extracellular vesicles, first‐in‐human intracochlear EV‐therapy, hearing loss, immunomodulation, Menière's disease, vesicle‐enriched secretome fraction

## Abstract

Extracellular vesicles (EVs) derived from the secretome of human mesenchymal stromal cells (MSC) contain numerous factors that are known to exert anti‐inflammatory effects. MSC‐EVs may serve as promising cell‐based therapeutics for the inner ear to attenuate inflammation‐based side effects from cochlear implantation which represents an unmet clinical need. In an individual treatment performed on a ‘named patient basis’, we intraoperatively applied allogeneic umbilical cord‐derived MSC‐EVs (UC‐MSC‐EVs) produced according to good manufacturing practice. A 55‐year‐old patient suffering from Menière's disease was treated with intracochlear delivery of EVs prior to the insertion of a cochlear implant. This first‐in‐human use of UC‐MSC‐EVs demonstrates the feasibility of this novel adjuvant therapeutic approach. The safety and efficacy of intracochlear EV‐application to attenuate side effects of cochlea implants have to be determined in controlled clinical trials.

## 1 INTRODUCTION

Hearing loss is the most common neurodegenerative disorder in man affecting more than 450 million people worldwide (Chadha et al., 2018). A majority of sensorineural impairment is caused by loss of hair cells and the consecutive degeneration of spiral ganglion neurons (Lawner et al., 1997). For many of the severely affected patients, treatment with cochlear implantation is required. The cochlear implant consists of an electrode array that is inserted into the cochlea. By direct electrical activation of the auditory nerve, the damaged hair cells are bypassed to elicit a hearing sensation that can result in appropriate speech understanding. However, cochlear implant patients have difficulty with hearing in noisy environments (Wilson & Dorman, 2008) and with music perception (McDermott, 2004). The electrode implantation itself can damage the already diseased inner ear. Surgical opening of the cochlea and insertion trauma may cause a foreign body reaction and acute or chronic inflammation with recruitment of leukocytes and expression of pro‐inflammatory cytokines (Bas et al., 2015; Seyyedi & Nadol, 2014). Tissue remodelling leading to fibrosis, loss of hair cells and degeneration of auditory neurons (Ishai et al., 2017; Quesnel et al., 2015) can result in loss of residual hearing (Kopelovich et al., 2015; Skarżyńska et al., 2018; Wanna et al., 2015). Currently, the only adjuvant therapy available are glucocorticoids which have limited efficacy (Causon et al., 2015; Cho et al., 2016; Nguyen et al., 2016; Rajan et al., 2012; Sweeney et al., 2015). Effective treatments to attenuate inflammation associated with cochlear implantation present an unmet clinical need.

Mesenchymal stromal cells (MSC) release immunomodulatory, anti‐inflammatory and neuroprotective factors (Al Jumah & Abumaree, 2012; Lescaudron et al., 2012; Li et al., 2012; Zhang et al., 2013). It is increasingly accepted that MSC can mediate tissue repair through secreted soluble and particulate factors. In 2007, a central role of extracellular vesicles (EVs) in tissue protection and repair was revealed by the use of MSC‐conditioned media in a myocardial infarction model (Timmers et al., 2008). This seminal study introduced the concept of organ repair via secreted vesicles of stromal cells. These findings soon were confirmed by various reports that demonstrated protection and regeneration by application of MSC‐EVs after cardiac and renal tissue injury (Bruno et al., 2009; He et al., 2012; Lai et al., 2010). Observations of improved post‐stroke neuroregeneration and prevention of post‐ischemic immunosuppression (Doeppner et al., 2015) as well as the attenuation of neuroinflammation and scarring after spinal cord injury (Romanelli et al., 2019) consolidated the proposition of MSC‐EVs as promising candidates to treat neurodegenerative disorders. We and others have shown that the immunomodulatory activity of MSC can be mediated in part by their secreted EVs (D'arrigo et al., 2019; Pachler et al., 2017; Yáñez‐Mó et al., 2015). MSC‐EVs exhibited a level of in‐vitro potency comparable to their intact parental cells. We have also demonstrated that MSC‐EVs derived from umbilical cord tissue (UC‐MSC‐EVs) exerted immunomodulatory activity on microglial cells (Warnecke et al., 2020). Spiral ganglion neuron survival in vitro was significantly improved in the presence of UC‐MSC‐EVs that were manufactured and characterized according to good manufacturing practice (GMP). The local application of UC‐MSC‐EVs to the inner ear attenuated hearing loss and protected auditory hair cells from noise‐induced trauma in a clinically relevant mouse model. Based on these preclinical data we hypothesized that UC‐MSC‐EVs can exert therapeutic effects in the inner ear and may attenuate inflammation elicited by insertion trauma or foreign body reaction. We therefore prepared for an individual experimental treatment on a ‘named patient basis’ with the aim to reduce cochlear implant related inflammation by using UC‐MSC‐EVs. One patient suffering from bilateral hearing loss due to Menière's disease received cochlear implantation in 2014 and was provided with an identical cochlear implant on the contralateral side in 2018 combined with the intracochlear application of UC‐MSC‐EVs. Evaluation of implant performance during the 24‐month follow‐up period reveals an initial safety profile for both the delivery procedure during surgery and the use of UC‐MSC‐EVs as a promising investigational medicinal product. To our knowledge, this local application in the course of cochlear implantation is the first‐in‐human treatment using allogeneic UC‐MSC‐EVs.

## 2 METHODS

### 2.1 Preparation and quality control of human umbilical cord mesenchymal stromal cell‐derived extracellular vesicles (UC‐MSC‐EVs)

**FIGURE 1:**
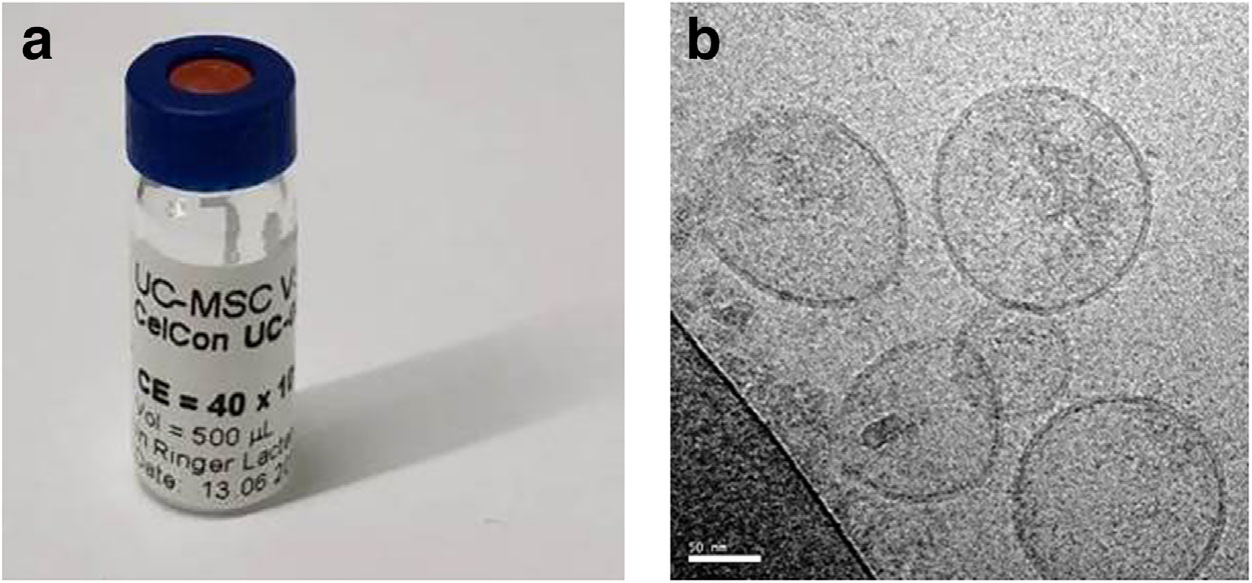
Therapeutic umbilical cord MSC‐derived extracellular vesicles (UC‐MSC‐EVs). (a) Filled and finished suspension containing 1.03 × 10^11^ UC‐MSC‐EVs/ml and 3.7 mg/ml protein in Ringer's lactate. (b) Cryo transmission electron microscopic image of UC‐MSC‐EVs, inserted white bar length equals 50 nm

After primary isolation of umbilical‐cord (UC) derived MSCs from a single donor, cells were cultured in fibrinogen‐depleted culture medium at 5% CO_2_ and 37°C as previously described (Warnecke et al., 2020). For the EV manufacturing process we have established a Master and a working cell bank from primary UC‐MSCs. At the time of harvest of the conditioned medium from the working cell bank, the MSCs are in passage 8, which corresponds to a total of 17 population doublings. In brief, no xenogenic substances have been employed throughout the entire manufacturing process and MSCs were expanded in the presence of human platelet lysate (Gimona et al., 2017; Laner‐Plamberger et al., 2015; Pachler et al., 2017; Rohde et al., 2019). The EV‐batch used for the preclinical experiments was extensively characterized in Warnecke et al., 2020 (Warnecke et al., 2020) and is identical to the one used for the patient application in this report. UC‐MSC‐EVs were prepared from conditioned medium by tangential flow filtration (TFF) and diafiltration, respectively, using a 100 kDa hollow fibre filter (Spectrum Labs). Ultimately, EVs were isolated by ultracentrifugation, the pellets were resuspended in Ringer's Lactate and the resulting solution was filtered through a 0,22 μm filter prior to storage at ‐80°C (Laner‐Plamberger et al., 2015; Pachler et al., 2017; Rohde et al., 2019). The clinical grade EV‐suspension was prepared in a pharmaceutically certified manufacturing site that operates according to good manufacturing practice (GMP) at the Paracelsus Medical University, Salzburg, Austria. This GMP unit has obtained a European manufacturing and distribution licence for the human investigational medicinal product ‘MSC‐EV’ granted by the Austrian regulatory authorities. Rigorous and standardized characterization of UC‐MSC‐EVs was performed as published previously (Warnecke et al., 2020) and complied with the criteria of the MISEV2018 guidelines (Théry et al., 2018) where applicable for therapeutic preparations. The ready to use suspension of UC‐MSC‐EVs was stored at – 80°C and delivered to the operating room as a sterile solution in 500 μl vials (Figure 1a. All clinical grade UC‐MSC‐EV batches were tested for the presence of endotoxins, bacterial sterility and the presence of mycoplasma. The identity, purity, potency and general safety parameters of the UC‐MSC‐EVs were characterized according to the established product release matrix of our manufacturing unit (Gimona et al., 2017; Pachler et al., 2017; Rohde et al., 2019). In short, quality control and potency parameters confirmed the previously published profile of UC‐MSC‐EVs. The preparation of the batch that was used for injection contained 1.03 × 10^11^ particles/ml with a diameter range of 110–130 nm as measured by nanoparticle tracking analysis (NTA, Model PMX110 from ParticleMetrix, Germany, **Figure S1** Nanoparticle Tracking Analysis of UC‐MSC‐EVs). Surface profiling by multiplex flow cytometry demonstrated the presence of the tetraspanins CD9, CD63, CD81, as previously described (Warnecke et al., 2020) as well as MSC‐EV markers CD29, CD44, CD49e and CD73 and revealed the absence of CD1/2/3/8/11c/14/19/20/24/25/31/40/45/56/69/86/133/142/209/326 and absence of the marker molecules HLA‐ABC, HLA‐DR, ROR1, SSEA‐4 (**Figure S2** Multiplex Marker Profiling of UC‐MSC‐EVs). Cryo‐transmission electron microscopic imaging confirmed the presence of UC‐MSC‐EVs by visualization of double‐layer lipid membranes around spherical objects (Figure 1b). Total protein amount of the used UC‐MSC‐EVs was 3.7 mg/ml. The biological activity of this particular clinical‐grade EV preparation was confirmed through *in vitro* and *in vivo* testing using inner ear cell culture and a noise trauma mouse model (Warnecke et al., 2020).

### 2.2 Patient demographics

One patient (male; age at implantation: 55 years) received allogeneic UC‐MSC‐EVs concurrent with left‐sided cochlear implantation in November 2018. Four years earlier, he was implanted in the contralateral ear with a MED‐EL Synchrony cochlear implant with a FLEX28 electrode array (MED‐EL Elektromedizinische Geräte GmbH, Innsbruck, Austria). The patient suffered from bilateral definite Menière's disease based on the diagnostic criteria defined and revised in 2015 (Lopez‐Escamez et al., 2015). When his second (left) ear lost hearing due to Menière's disease, he was offered cochlear implantation with the identical device (MED‐EL Synchrony FLEX28) in combination with delivery of UC‐MSC‐EVs to prevent potential insertion trauma related side effects. The patient agreed to this individual treatment using adjuvant intracochlear UC‐MSC‐EV injection during the surgical procedure on a ‘named patient basis’.

### 2.3 Surgical procedure including UC‐MSC‐EV application

**FIGURE 2:**
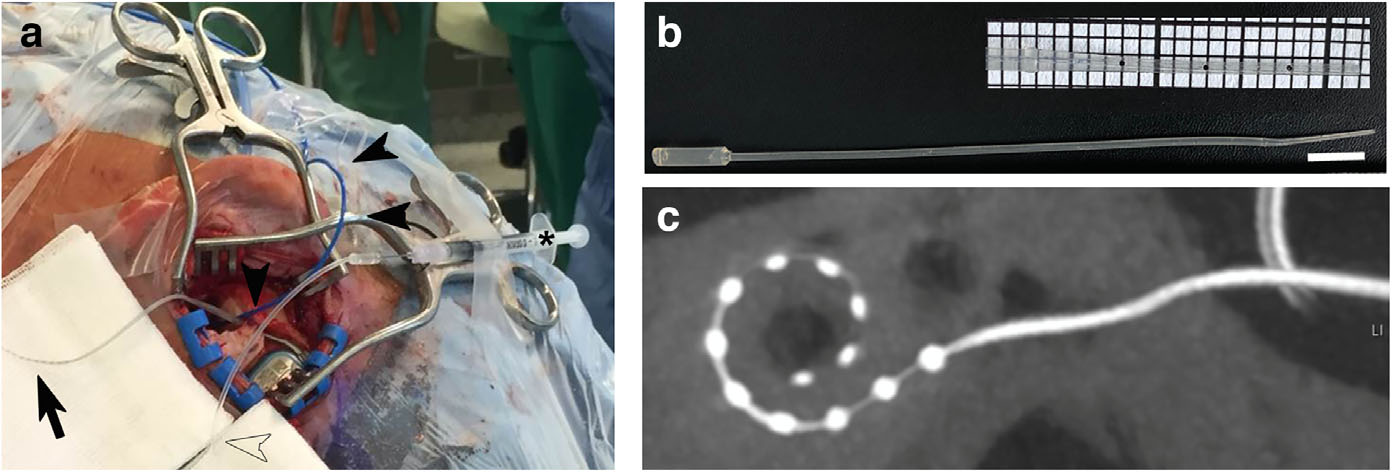
Surgical procedure and intraoperative UC‐MSC‐EV application. (a) Intraoperative image showing the mastoid with the electrode array prepared to be inserted (black bold arrow). Electrocochleography recordings were performed with an extra electrode (black arrow heads). The inner ear catheter (hollow arrow head) is attached to a syringe (asterisk) and contains the EV solution. (b) MED‐EL inner ear catheter with close up of the tip (insert). The tip has three marking spaced 5 mm apart to allow insertion to a predicted depth into the inner ear. Bar equals 1 cm. (c) Postoperative cone beam‐computed tomography showing the intracochlear position of the electrode array of the vesicle‐treated side

Both the left‐ and the right‐sided cochlear implantations were performed using the identical electrodes by experienced surgeons at the department of Otorhinolaryngology, Hannover Medical School and according to international cochlear implant standards. We performed all procedures via a round window approach. Figure 2a depicts the surgical approach with the inner ear catheter, the implant and the measurement electrode for electrocochleography in place. The catheter (Figure 2b) and its intraoperative use have been previously described (Prenzler et al., 2018). The catheter was prefilled with the suspension containing the UC‐MSC‐EVs and fixed near the site of the mastoidectomy (Figure 2a). The cochlear implant was placed in the mastoid bed. Extracochlear electrocochleography recordings for monitoring of cochlear function during insertion were performed as described in detail previously (Haumann et al., 2019).

For EV application, the vial was transported on ice to the operating room and was allowed to thaw at room temperature for 20 min. One hundred μl of UC‐MSC‐EV suspension was drawn up with a 1 ml syringe. The syringe was connected to the inner ear catheter as previously described (Prenzler et al., 2018) and the suspension was slowly injected into the cochlea until an outflow of the fluid was observed at the insertion site. The total delivery volume was estimated to range from 20 to 40 μl corresponding to a dose of 2 × 10^9^ to 4 × 10^9^ UC‐MSC‐EVs in total for local application. As the concentration of the solution was determined as 1 × 10^11^ particles/ml by NTA, a total of roughly 1 × 10^8^ particles/μl could be applied in a volume between 20 and 40 μl which was retained in the cochlea. The catheter was then slowly removed from the cochlea while continuing gentle EV suspension delivery. Subsequently, the MED‐EL Synchrony FLEX28 electrode was inserted. Cone beam – computed tomography (CB‐CT) was performed to verify the correct intracochlear position of the electrode array (Figure 2c).

### 2.4 Audiological evaluation

Electrocochleography recordings prior to the opening of the round window and during insertion were performed to monitor the cochlear functionality. After electrode insertion, impedance measurements were performed using the standard MED‐EL telemetry system (MAX interface box, clinical software Maestro 6 or later) on all 12 electrode contacts. The audiological evaluation performed for first fitting (FF) was done 4 weeks after cochlear implantation over a time course of 1 week and consisted of the Freiburg monosyllable test (FBM), the Hochmair‐Schulz‐Moser (HSM) sentence test and the Oldenburger sentence test measured in noise (OLSA). Scores for the treated ear were compared to similar measurement time points for his contralateral implanted ear.

### 2.5 Ethics

All procedures were performed in accordance to the ethical principles for medical research in humans (Declaration of Helsinki). The patient gave a written informed consent after in‐depth consultation regarding the first‐in‐human use and the possible risks and potential complications of the named patient use treatment (e.g., tumor induction, meningitis and ossification of the cochlea). The use of the UC‐MSC‐EVs was reported to, and the use of the inner ear catheter for EV‐delivery as well as the performance of electrophysiological measurements were approved by the local ethics committee (Hannover Medical School, reference numbers 2740‐2015 and 3279‐2016, respectively).

### 2.6 Data analysis

Graphical analysis was performed with Origin Version 9.1. The data were validated by using one‐way ANOVA followed by Bonferroni's multiple comparison test. P values of less than 0.05 were considered statistically significant. Levels of significance are indicated as * *P* < 0.05 and *** *P* < 0.001.

## 3 RESULTS

We have previously demonstrated the potential of clinical grade UC‐MSC‐EVs to inhibit T‐cell growth and to downregulate gene expression of pro‐inflammatory factors (Pachler et al., 2017; Romanelli et al., 2019). In addition, the neuro‐ and otoprotective effects of EVs *in vitro* and *in vivo* as well as the lack of toxicity in hearing mice were demonstrated recently (Warnecke et al., 2020). These results encouraged us to apply UC‐MSC‐EVs in an individual patient treatment performed on a ‘named patient basis’ during cochlear implantation in an effort to reduce inflammation caused by the electrode insertion.

During surgery, the preserved on‐going stimulus response recordings of electrocochleography for the left inner ear demonstrated that UC‐MSC‐EV infusion had not altered the residual inner ear function. Within 5 days after surgery and during a follow up period of more than 24 months, no signs of acute systemic or local toxicity such as fever or allergic reactions were noticed. Most importantly, speech intelligibility improved within the first year and stayed constant for the second year after implantation.

Speech understanding pre‐ and post‐implantation of both ears over a time course of 2 years is listed in the Table 1 ‘Speech Perception’. Speech intelligibility with the HSM test in noise at a SNR of 10 dB was 65% for the UC‐MSC‐EV‐treated left side at first fitting (FF). At 3 months after FF, speech understanding in noise (10 dB SNR) increased to 85% (UC‐MSC‐EV‐treated) and reached comparable results at 12 and 24 months for both sides, representing a ceiling effect. In the more difficult testing condition with a signal to noise ratio of 5 dB, speech intelligibility at 12 and 24 months were 56% and 64% for UC‐MSC‐EV‐treated side and 49% and 37% for the contralateral ear. With the OLSA (Oldenburg sentence test), the patient achieved 50% intelligibility at ‐0.7 and ‐1.6 dB SNR after 12 and 24 months, respectively, with the vesicle‐treated side and ‐2.0 dB SNR with the non‐EV‐treated ear.

**TABLE 1:** ‘Speech perception’: Speech intelligibility after cochlear implantation with or without local application of UC‐MSC‐EVs

Preoperative FBM (unaided, headphones, dB optimal)		
	Left	Right
	110 dB (15%)	110 dB (65%)
Postoperative FBM		
Month	Left, EVs	Right, control
FF	50%	80%
3	55%	65%
6	75%	65%
12	80%	90%
24	90%	95%
Postoperative HSM 10 dB SNR		
Month	Left, EVs	Right, control
FF	65%	37%
3	85%	69%
6	85%	60%
12	89%	81%
24	90%	85%
Postoperative HSM 5 dB SNR		
Month	Left, EVs	Right, control
FF	25%	n.d.
3	41%	n.d.
6	62%	n.d.
12	56%	49%
24	64%	38%
Postoperative OLSA 50%		
Month	Left, EVs	Right, control
12	‐0.7 dB SNR	‐2.0 dB SNR
24	‐1.6 dB SNR	n.d.

Abbreviations: dB, decibel; EVs, Extracellular vesicles derived from umbilical cord‐mesenchymal stromal cells; FBM, Freiburger monosyllabic test; FF, first fitting; HSM, Hochmair‐Schulz‐Moser sentence test; n.d., not determined; OLSA, Oldenburg sentence test; SNR, signal to noise ratio.

**FIGURE 3:**
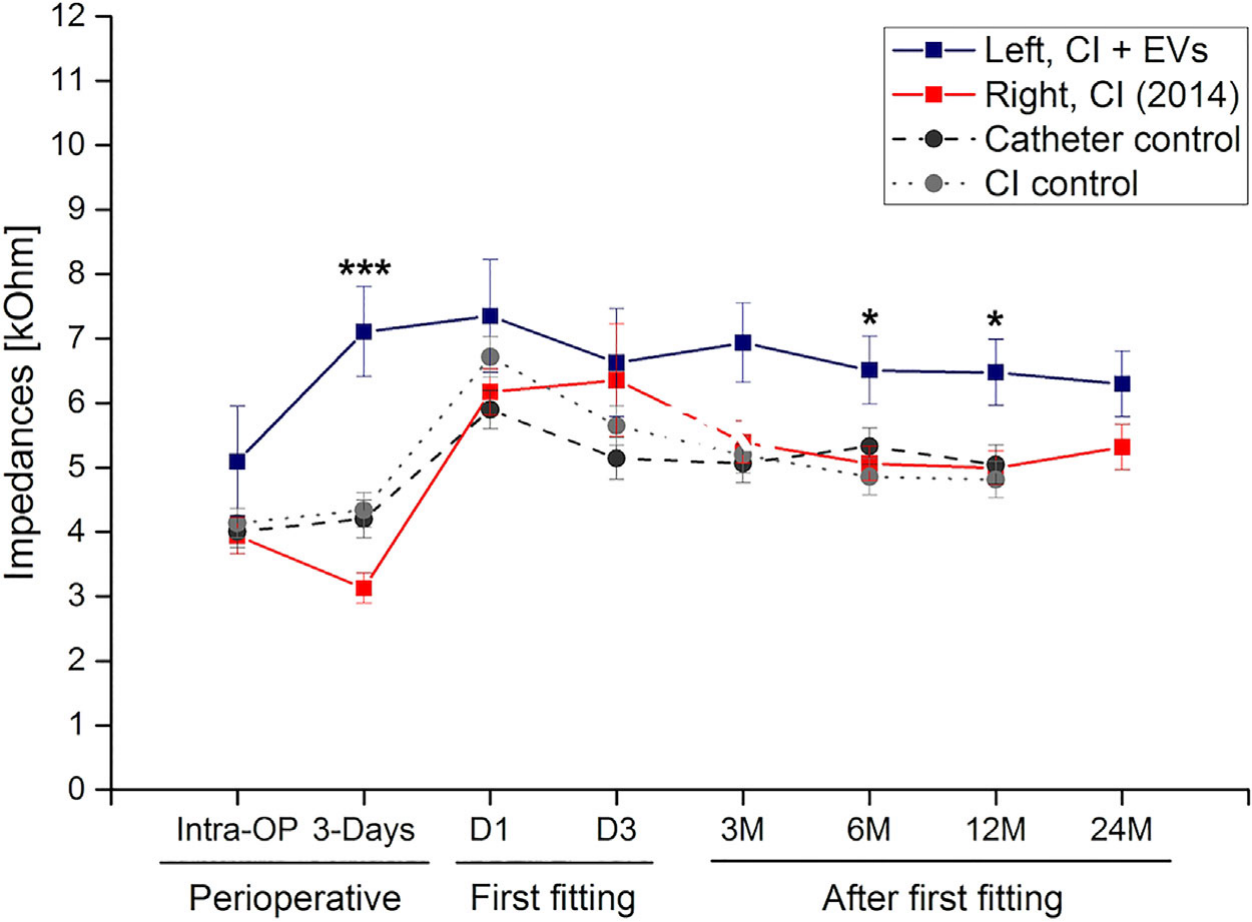
Mean impedance values over time. Following bilateral cochlea implantation (CI) at the right side first and the contralateral side 4 years later, mean impedance values are depicted over time. Blue line depicts the vesicle treated side and red the patient's contralateral side. Historical data from controls (dotted line) and steroid‐treated patients via the inner ear catheter (intermitted line) are included for comparison. Impedance values were recorded for each of the twelve electrode contacts per side and condition and are depicted as mean values and standard deviation over a time course of more than 24 months. The first fitting (FF) is done 4 weeks after cochlear implantation. Data from day 1 and day 3 during FF are shown as well as data from the follow‐up visits (3 M, 6 M, 12 M and 24 M). Levels of significance are indicated as * *P* < 0.05 and ***P < 0.001

The course of individual impedance values for each of the twelve electrode contacts are depicted in Figure 3 and show measurements at 4 weeks after electrode implantation (i.e. time point for FF) as well as 3, 6, 12 and 24 months after FF. Impedance values represent the electrical resistance of the individual implant electrode contacts. These are measured as the ratio ‘voltage between the two electrodes’ divided by ‘injected current’ (Neuburger et al., 2009). The mean impedances in the EV‐treated left side were significantly higher when compared to the right side receiving intravenous steroid treatment 4 years ago for the values measured at 3 days post implantation and 6 as well as 12 months after FF. Compared to mean impedance values from a previously published study on patients treated with steroids applied via the inner ear catheter and a non‐treated control group (Prenzler et al., 2018), significant differences were also found when comparing the EV‐treated side (except for the 6 months data of the steroid catheter).

## 4 DISCUSSION

This present report provides the first evidence of the feasibility for the application of allogeneic human MSC‐EVs into the inner ear. Our goal was to limit the potential damage to the inner ear from a foreign body reaction and inflammation induced by the opening of the cochlea and by the insertion of the electrode array. Dampening of the implantation‐induced inflammation can lead to reduced scarring and ossification, resulting in improved cochlear health and speech perception (Fujioka et al., 2014).

During a 5‐day observation period following the intracochlear EV‐injection, no signs of acute systemic or local toxicity such as fever or allergic reactions were noted. Most importantly, the 24‐month follow‐up period showed stability of speech intelligibility after initial improvement. These observations suggest that the local injection of allogeneic UC‐MSC‐EVs to the inner ear is safe in principle. However, the experience from one single patient treatment is a limiting factor and is not sufficient for a robust safety assessment according to good clinical practice. No direct evidence could be provided by this single case observation that the EV‐injection resulted in reduction of inflammation or other beneficial effects. Nevertheless, these results mirror the existing experience with the application of MSCs in the central nervous system. Stromal cells have been tested in a large number of studies and their immunomodulatory potential in the context of neurodegenerative diseases has been shown in various preclinical studies (Al Jumah & Abumaree, 2012; Bas et al., 2014; Lescaudron et al., 2012; Li et al., 2012; Zhang et al., 2013). Evaluation of MSC therapy in septic shock models resulted in preclinical proof of concept (Lalu et al., 2012, 2016) and a first evidence of clinical safety (Mcintyre et al., 2018).

Clinicaltrials.gov lists more than 830 studies using ‘mesenchymal cells’ for a wide range of clinical conditions. Diseases treated with MSC range from graft‐versus‐host‐disease after bone marrow transplantation in haematological disorders, immunomodulation in solid organ transplantation, autoimmune diseases such as inflammatory bowel diseases, orthopaedic diseases such as painful degenerative disc disease, cardiomyopathy, acute respiratory distress syndrome, stroke and traumatic brain injury (Marquez‐Curtis et al., 2015). Among these clinical trials, more than 20 studies are currently listed for using umbilical cord MSC. Specifically, with respect to umbilical cord‐derived cells, data from various experimental treatments of more than 1900 patients have been published in over 90 clinical reports. These studies demonstrate a high safety profile of a cell therapy approach using UC‐MSC. However, only few studies have shown clinical efficacy. For example, more than 500 clinical trials using MSC from various sources for the treatment of different diseases have been registered in 2015 and less than 2% of these studies have published their outcomes. The reason for the lack of published efficacy data from clinical trials is yet unclear and is in contrast to the ample in vitro data that claim efficacy. After years of research, our understanding of the nature and function of MSC from various tissues has undergone a paradigm shift from cell‐based regeneration towards paracrine‐mediated tissue repair giving rise to the concept of exclusively using EVs instead of their viable parental cells (Phinney & Pittenger, 2017).

Intercellular communication via EVs represents a recently discovered mechanism by which cells cannot only exchange material and information but also mediate relevant function of the innate and adaptive immune systems (Malkin & Bratman, 2020). Furthermore, EVs have been shown to be potent mediators of the anti‐fibrotic effects of UC‐MSC (Malkin & Bratman, 2020).

Based on the confined total number of around 2 million cells in the human cochlea and on our own unpublished observations that a homeostasis of vesicle secretion and uptake is achieved at roughly 1000 EVs per cell in cell culture, we estimated that a dose of 2 – 4 × 10^9^ UC‐MSC‐EVs would be needed for local injection for the inner ear. Efficacy quantification of any adjuvant treatment in cochlear implantation is challenging despite having several tests that can be employed to measure residual cochlear function, cochlear health and speech intelligibility, reflecting the multiple factors that can influence outcomes (Carlson, 2020). For example, the overall vulnerability of the inner ear due to the disease may be one factor hampering the preservation of residual cochlear function. This is particularly the case in patients with Menière's disease, where high levels of the pro‐inflammatory cytokine interleukin‐1, characteristic for immune‐mediated inflammatory diseases, have been found in the peripheral blood (Lopez‐Escamez et al., 2018).

With the results obtained from preclinical safety and toxicity data in vivo (Warnecke et al., 2020) and from this first‐in‐human experimental treatment, we conclude that the intraoperative application of UC‐MSC‐EV preparations is a feasible procedure. During a follow‐up period of more than 24 months after implantation, we have not observed any adverse reactions or deterioration of speech performance after the treatment performed on a ‘named patient use’ basis. In fact, we observed an increased hearing capacity of the UC‐MSC‐EV‐treated side, even though the pre‐implantation speech understanding was significantly worse when compared to the contralateral ear at the time of implantation. Although these outcome parameters and the fact that we have observed no complications in this particular case are promising, a major shortcoming of this report is that the experience from one single patient treatment is not sufficient to draw any further conclusions regarding safety, potency or efficacy. The lack of measures to reliably demonstrate immunological responses after cochlear implantation in patients limits the quantification of anti‐inflammatory effects of EVs. Furthermore, the various immunological aspects of allogeneic EV injection have to be studied more extensively. Therefore, we are currently investigating immunotoxicity, autoimmune potential and pro‐inflammatory effects of local UC‐MSC‐EV application in animal models of cochlear implantation. For the initiation of a clinical phase 1 safety trial, these preclinical pharmacokinetic and toxicological analyses are mandatory.

After this first‐in human case report, a clinical phase 1 trial to test the safety of intracochlear UC‐MSC‐EV application is currently planned. Controlled phase 2 and phase 3 clinical trials will be required to evaluate the clinical benefit of the adjuvant local treatment using allogeneic umbilical cord‐derived MSC‐EVs in cochlear implantation.

## DISCLOSURE STATEMENTS

Eva Rohde: CEO of PMU Innovations GmbH (Salzburg) and Medical Consultant of MDimune Inc., (Seoul, Korea). Mario Gimona: Consulting Chief Manufacturing Officer, MDimune Inc., (Seoul, Korea).

## FUNDING

This work was funded by the Deutsche Forschungsgemeinschaft (DFG, German Research Foundation) under Germany's Excellence Strategy – EXC 2177/1 ‐ Project ID 390895286 and by the Government of Salzburg State under the WISS2025 Strategy –Project ID ‘ExtraNeu’.

## Supporting information

Supporting information.
