# Supplementary material for: First‐in‐human intracochlear application of human stromal cell‐derived extracellular vesicles
Source: J Extracell Vesicles. 2021 Jun 4;10(8):e12094. doi: 10.1002/jev2.12094 (PMC8178433; doi:10.1002/jev2.12094)

SUPPLEMENTAL MATERIALS AND METHODS

Nanoparticle tracking analysis (NTA) in light scatter mode

To determine the size and amount of particles in the individual umbilical cord-derived mesenchymal stromal cell-extracellular vesicle preparations (UC-MSC-EVs), samples were analyzed in light scatter mode in a Nanoparticle Tracking Device (ZetaView PMC 110 from Particle Metrix). Previously frozen EV preparations were used and samples were diluted to a concentration of 4 – 7 x 10^7 particles/mL in PBS. Prior to NTA analysis, the instrument was calibrated using Yellow/Green-labeled 100 nm polystyrene standard beads (1 : 1.000.000 dilution in ddH_2O). The minimum brightness was set to 20 arbitrary units (AU), temperature to 21.5 °C, shutter to 70 AU, and sensitivity to 85 AU. Subsequently, data for two exposures at 11 measurement positions were acquired per sample. Based on the Stokes-Einstein equation, particle size was calculated using the ZetaView software (PMX 110: Version 8.4.2).

MACSPlex surface protein profiling

The bead-based multiplexed FACS-based MACSPlex Exosome Kit (Miltenyi Biotec) is an assay for the analysis of surface markers present on UC-MSC-EVs. To characterize the various MSC-EV preparations we used the MACSPlex kit according to the manufacturer´s instructions and following a validated standard operating procedure with 5 x 10^7 to 5 x 10^8 total particles as input. Data acquisition was done using a FACS Canto II instrument (BD Biosciences). Data normalization was directed towards CD9/CD63/CD81 APC signal. Isotype control normalization was performed as described [Ref 33 of main manuscript].

SUPPLEMENTAL FIGURES

Figure S1 Nanoparticle Tracking Analysis of UC-MSC-EVs

(A) Nanoparticle Tracking Analysis (NTA, light scatter mode) reveals the size distribution of particles within the umbilical cord-derived UC-MSC-EVs with a median particle diameter shown.

ZetaView – PARTICLEMETRIX – www.particle-metrix.de

Electrophoresis & Brownian Motion
Video Analysis
Laser Scattering Microscopy

Operator (Report): ZetaView
Video Operator: ZetaView

Sample Parameters
Sample Name: EV_UC-MSC_LA0013-1_Celcon-UC-0007-medTFF-100kDTFF-und-UZ-13.06.18
Comment: Sample Remarks0:
Sample Remarks1:
Sample Remarks2:
Electrolyte: PBS
Temperature: 21,63 °C sensed
pH 7,0 entered
Conductivity: 15000,00 µS/cm sensed

Result (sizes in nm)

	Number	Concentration	Volume
Median (X50)	115,8	115,8	170,3
Span	51,9	51,9	76,3

Concentration: 6,1E+7 Particles / mL
Dilution Factor: 1700
Original Concentration: 1,0E+11 Particles / cm³

Quality
Average Counted Particles per Frame: 153
Number of Traced Particles: 1687

Measurement Parameters
Cell S/N: CA0079

Measurement Mode: Size Distribution 2 Cycles
11 Positions, 1 Removed for Analysis

Analysis Parameters
Max Size: 1000, Min Size: 5, Min Brightness: 20

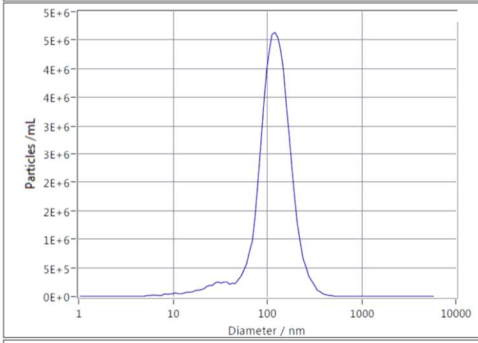

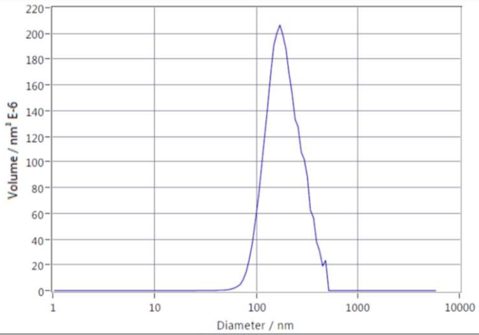

Peak Analysis (Concentration)

Diameter / nm	Particles/mL	FWHM / nm	Percentage
121,9	4,6E+6	99,0	100,0

X Values

	Number	Concentration	Volume
X10	66,6	66,6	108,8
X50	115,8	115,8	170,3
X90	180,7	180,7	293,3
Span	1,0	1,0	1,1
Mean	125,0	125,0	194,3
StdDev	51,9	51,9	76,3

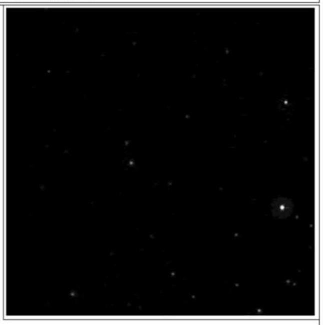

Comment

(Signature)

Analyzed Video: D:\NTA Berichte\20180621_0009_EV_UC-MSC_LA0013-1_Celcon-UC-0007-medTFF-100kDTFF-und-UZ-13.06.18_size.avi

ZetaVIEW S/N 197, Software ZetaView 8.03.08.03, Camera 0,703 µm/px

Experiment: 2018-06-21 12:36, Report: 2018-06-21 12:38

Figure S2 Multiplex Marker Profiling of UC-MSC-EVs

Surface profiling of UC-MSC-EVs by MACSplex multiplex assay confirms the presence of tetraspanins (CD9, CD63, CD81) typical for EVs in addition to CD29 (Integrin beta-1), CD44 (Receptor for hyaluronic acid), CD49e (Integrin alpha-5) and melanoma-associated chondroitin sulfate proteoglycan (MCSP) and the absence of CD1/2/3/8/11c/14/19/20/24/25/31/40/45/56/69/86/133/142/209/326 and of the marker molecules HLA-ABC, HLA-DR, ROR1, SSEA-4.

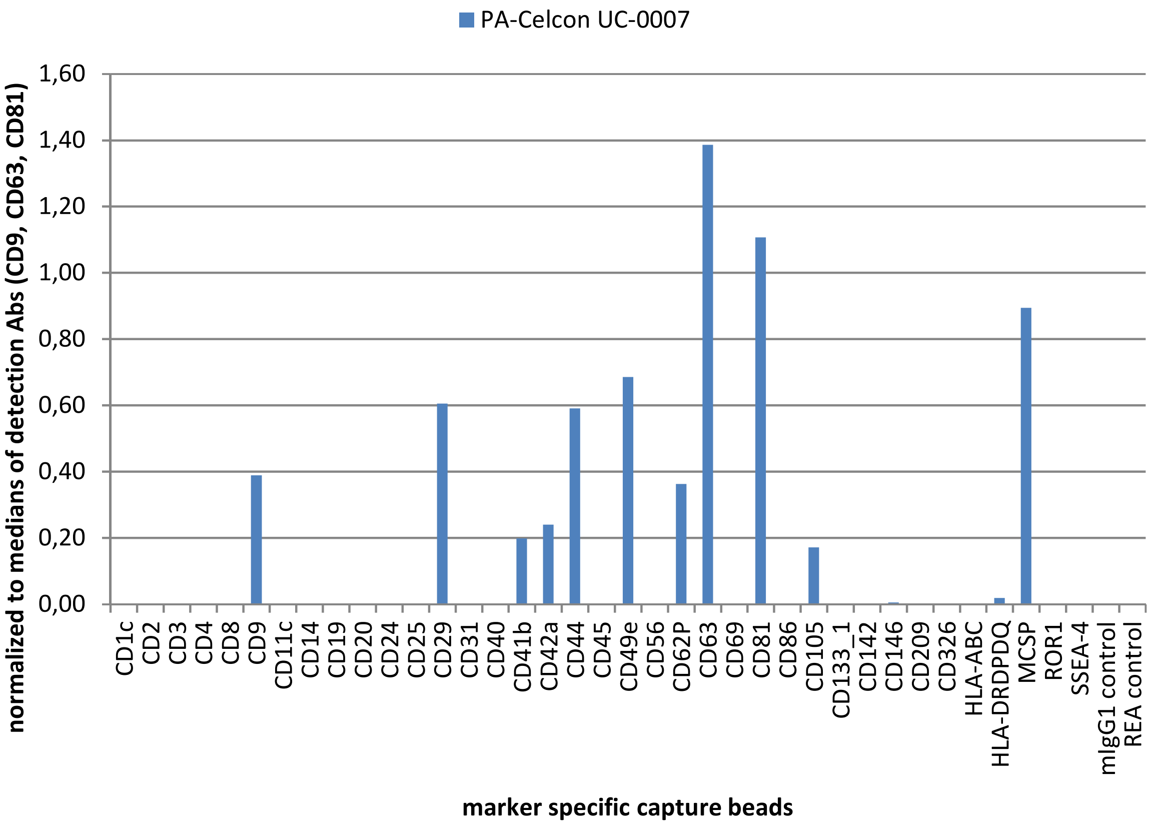

Supplement: Supplementary file 1 — Supporting information. [file JEV2-10-e12094-s001.pdf]
